# The power of peers: Design‐based research on stimulating peer‐assisted learning for enhancing the clinical‐reasoning learning process in the workplace

**DOI:** 10.1111/medu.15613

**Published:** 2025-02-14

**Authors:** Larissa I. A. Ruczynski, Bas J. J. W. Schouwenberg, Cornelia R. M. G. Fluit, Marjolein H. J. van de Pol

**Affiliations:** ^1^ Research on Learning and Education, Radboudumc Health Academy Radboud University Medical Center Nijmegen Netherlands; ^2^ Department of Pharmacy, Division Pharmacology‐Toxicology Radboud University Medical Center Nijmegen the Netherlands; ^3^ Department of Internal Medicine Radboud University Medical Center Nijmegen Netherlands; ^4^ Department of Primary and Community Care Radboud University Medical Center Nijmegen Netherlands

## Abstract

**Introduction:**

Peer‐assisted learning (PAL) is widely used in undergraduate medical education, and collaborative learning is gaining momentum. Unfortunately, literature shows that students utilise their peers less during undergraduate clerkships, a phase when PAL is known to be particularly useful to students and their clinical‐reasoning learning process. For this reason, we investigated the following question: How can we design a workplace for undergraduate students that fosters PAL with regard to enhancing their clinical‐reasoning learning practice?

**Methods:**

We used a design‐based research (DBR) methodology, involving iterative development to address a complex educational issue. Between September 2022 and October 2023, stakeholders participated in four work conferences (WCs). The final output consists of design principles for a workplace to encourage PAL for the enhancement of the clinical‐reasoning learning practice for undergraduate students, as well as an evaluated 2‐month pilot intervention, ‘paired consultation’, that aligns with these design principles. This study is conducted in the Netherlands.

**Results:**

A total of 101 individuals participated in this study. In the WCs, design principles were continuously discussed, while an intervention was developed under the guidance of the research group. Discussion topics included reciprocal vulnerability, culture, autonomy, flexibility, ownership, psychological safety, competition and the role of supervisors. Overall, the pilot was considered a success by all involved and small adjustments were made throughout. Students mentioned that observing peers supported their clinical‐reasoning learning process. Supervisors expressed growing enthusiasm for PAL.

**Conclusion:**

To ensure successful interventions based on the design principles, three topics deserve attention: (1) Students must share responsibility for learning with peers, (2) supervisors must contribute to the evolution towards a collaborative learning environment and (3) a community of learners within a community of practice can enhance the collaborative learning practice. Further research should focus on guiding the workplace in this transition towards collaborative learning.

## Abstract 2

Do you want to know how to design a workplace that fosters peer‐assisted learning for the enhancement of the clinical‐reasoning by undergraduate students? Find out in this paper!

## 1 INTRODUCTION

Clinical reasoning is one of the key competencies of a physician. Almost all actions undertaken by physicians in their daily routine can be considered a component of the clinical‐reasoning process.^1^ Historically, clinical reasoning was considered an individualistic process of each physician; however, over the past decade, there has been a shift towards patient care in general and clinical reasoning in particular being considered more as a team effort.^2^, ^3^ Despite this change, the focus of our medical curricula still lies primarily on the individual in both the education and assessment of clinical reasoning, even when students participate in group learning activities.^2^, ^4^, ^5^ At the same time, health care is increasingly under pressure: Patients have shorter hospital stays despite presenting with more complex conditions, other health care professionals require on‐the‐job training and medical specialists have less time for teaching responsibilities, reducing their availability for students. This can lead to a reduction in learning opportunities for students in practice and prompts us to consider alternative ways to shape students' learning journeys in practice, while keeping in mind that they will always be in close proximity to one another.

When students engage in collaborative learning with and from each other, it is referred to as peer‐assisted learning (PAL).^6^ Formally, PAL can be defined as ‘students, who are not professional teachers, teaching each other something and, by doing so, also learning themselves’.^7^ Various forms of PAL have been identified and studied in the clinical‐reasoning learning practice,^8^ with undergraduate medical students already using PAL in clinical practice^6^, ^9^, ^10^, ^11^ to develop their clinical‐reasoning skills.^8^, ^12^ Whereas PAL was traditionally understood as a tutor–tutee structure with a vertical transfer of knowledge, PAL is now perceived more flexibly in workplace settings with students often being each other's equal sparring partners.^8^ This can be considered an integral part of collaborative learning,^13^ involving both the co‐regulation and socially shared regulation of learning.^4^, ^14^ Bransen et al. describe the difference in the co‐regulation and socially shared regulation of learning as the latter being more reciprocal.^4^, ^15^, ^16^ This contributes to the recent development that PAL should be more about learning *with* peers rather than learning *from* peers.^3^

The added value of PAL has been described in the literature. In addition to improving academic and clinical performance,^9^, ^17^ PAL can play a role in fostering the development of skills related to professional identity formation (PIF), self‐directed learning and lifelong learning.^16^, ^18^, ^19^, ^20^, ^21^ Previous research indicates that undergraduate students in the clinical clerkships phase value their peers as role models, discussion partners and sources of support during the development of their clinical‐reasoning skills.^8^ They also utilise their peers' clinical‐reasoning skills as a complementary resource for cognitive growth.^8^, ^18^ Because students operate on a comparable level, they can recognise each other's difficulties and provide effective assistance. While a supervisor may be considered essential at some point in their peer‐to‐peer learning process, students can make significant progress independently first.^8^, ^20^

Using PAL to establish collaborative learning during the development of clinical‐reasoning skills demands a change in the role of the supervisor as well.^22^, ^23^, ^24^ Traditionally, the development of clinical‐reasoning skills in practice revolved around cognitive apprenticeships with supervisors^25^; however, today, these relationships are strained due to time pressure and increasing workloads.^18^, ^19^, ^26^ PAL can be used as a supplement to cognitive apprenticeships in these situations.^25^ This requires a critical assessment of which aspects of clinical reasoning should be acquired from a supervisor and which can be effectively learned among peers. While PAL may not necessarily save them time, supervisors can now utilise the time they spend supervising students in a more fulfilling manner, such as by concentrating on facilitating meaningful discussions on clinical reasoning.^18^

That being said, the literature also shows us that, oddly enough, students utilise their peers less during undergraduate clerkships,^15^, ^16^ a phase when PAL is known to be particularly useful to students.^9^ One could question whether this regression in the utilisation of PAL during undergraduate clerkships is favourable, especially when PAL contributes to the development of clinical‐reasoning skills and several competencies linked to clinical reasoning.^4^, ^8^, ^9^, ^10^, ^11^, ^16^, ^17^, ^19^, ^27^, ^28^

One of the findings from our previous research highlights the significant role of context (e.g. environment, objects, time and actors) in the occurrence of PAL in the workplace. Context, along with the individuals involved, can either facilitate or hinder student learning by influencing the available space and students' sense of autonomy in organising their learning activities.^29^ This, in turn, has an impact on the prevalence of PAL. We therefore believe that, to encourage PAL, a conducive learning environment must be established.

For this reason, in the present study, we investigated the following question: How can we design a workplace for undergraduate students that fosters PAL with regard to enhancing their clinical‐reasoning learning practice? In pursuit of answers, we undertook design‐based research (DBR),^30^, ^31^, ^32^, ^33^ simultaneously developing an intervention and devising design principles. Following the DBR tradition, the entire process unfolded within the context of the clinical practice and in collaboration with its stakeholders.

## 2 METHODS

### 2.1 Research setting

**FIGURE 1:**
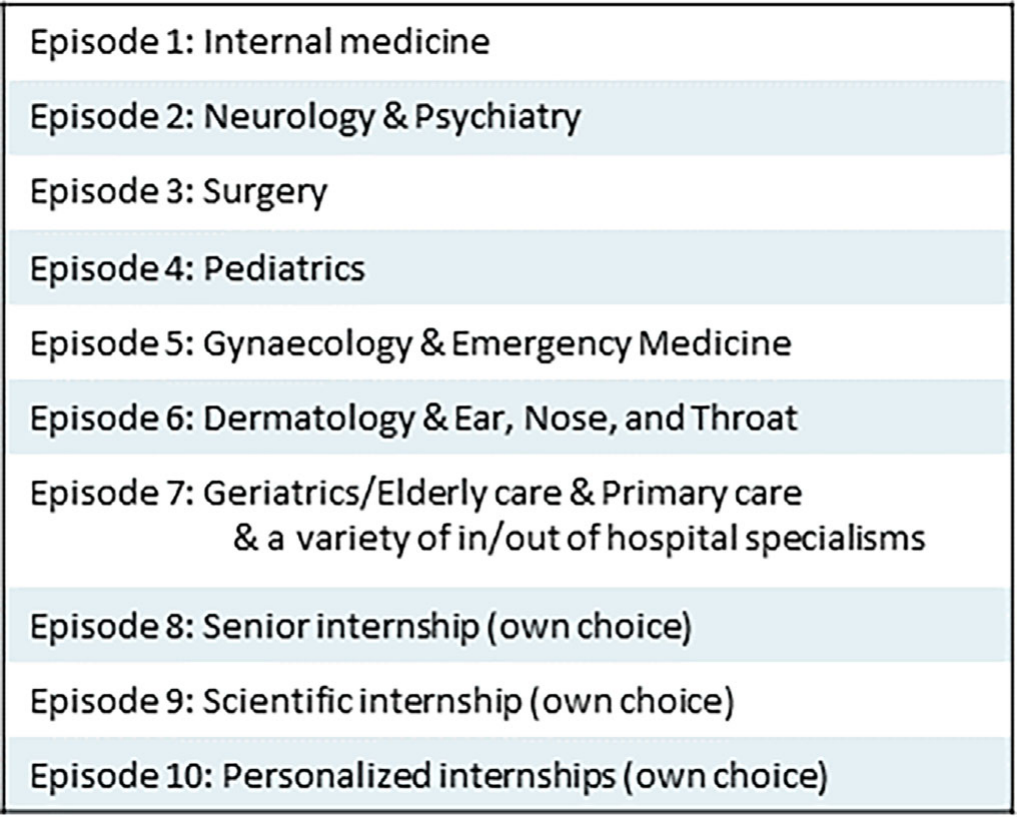
Overview of clerkships within the master's curriculum at Radboudumc. [Color figure can be viewed at wileyonlinelibrary.com]

This study took place at the Radboud University Medical Center (Radboudumc) and the affiliated Canisius Wilhelmina Hospital (CWZ), both located in Nijmegen, the Netherlands. Radboudumc is an academic hospital with a medical faculty, while CWZ is a community hospital. Our medical education program spans over 6 years, encompassing a 3‐year bachelor's program followed by a 3‐year master's program. Within the master's program, students engage in a series of 10 clerkships within the Radboudumc or one of its affiliated hospitals, with the first eight clerkships being completed in a fixed sequence (Figure 1). Preceding and succeeding each clerkship, formal educational modules are conducted at the medical faculty. During the final academic year, students exercise the option to choose their preferred departments for the completion of their clerkships.

### 2.2 Research population

The study population comprises undergraduate medical students enrolled in the master's program at Radboud University, who are both the focus of the study and participants in the work conferences (WCs). Purposive sampling was used to assemble an inclusive group of stakeholders for the WCs with diverse perspectives that would enrich the WC discussions. Various factors were considered, including departments (specialty/surgical departments), in‐hospital and primary care professions, professional roles (medical specialist, resident, coordinator, educationalist, researcher), clerkship duration (2–8 weeks) and ensured patient advocates, and near‐graduates were also included. WC2, however, was an open workshop during a yearly conference of the Netherlands Association of Medical Education (NVMO). Although purposive sampling was not feasible, stakeholders, as described above, were explicitly invited to participate in the workshop. The head researcher (LR) initiated and maintained contact with possible participants and contacted them for inclusion. If someone was not available as a stakeholder for the WCs, they were asked to provide a suitable replacement candidate that LR could contact. We explicitly informed participants at the start of our WCs that their contributions to this research would be acknowledged equally. We encouraged everyone to use their unique backgrounds to enrich the discussions that lay ahead.

### 2.3 Research group

The research group comprised three medical doctors (MvdP, BS and LR) with various expertise (primary and elderly care, internal medicine, clinical pharmacology and emergency medicine) and one educationalist with a medical background (CF). All researchers are active within the undergraduate curriculum as program director (MvdP), clinical supervisor (BS and MvdP) and/or educator (MvdP, BS, LR and CF).

### 2.4 Ethical considerations

Ethical approval was granted by the Ethical Review Board of the NVMO, dossier number 2022.1.10. All participants were provided with comprehensive information regarding their involvement well in advance. Prior to commencing data collection, LR obtained informed consent from each participant. Data collection and processing throughout the WCs were conducted anonymously, with no personal information collected beyond what was provided in the informed consent forms. During each pilot evaluation interview, data were gathered and pseudonymised by LR, who took notes of the interviews. These notes were presented to the participants to be checked and, if necessary, modified. Within the summary of these pilot evaluation notes, participants were fully anonymised.

### 2.5 Research design

We used a DBR methodology,^30^, ^31^, ^32^, ^33^ consisting of an iterative development process where solutions are devised for a practical and complex educational issue.^31^ Conducting a DBR necessitates gaining a comprehensive understanding of a problem before conceiving and implementing potential solutions^33^; thus, DBR offers a framework for empirical research that generates theoretical insights applicable for informing others,^31^, ^33^ thereby bridging the gap between research and practice.^30^, ^31^ The final output of this DBR will consist of design principles for a workplace to encourage PAL for the enhancement of clinical‐reasoning skills among undergraduate students, as well as an evaluated pilot intervention that aligns with these design principles.

**FIGURE 2:**
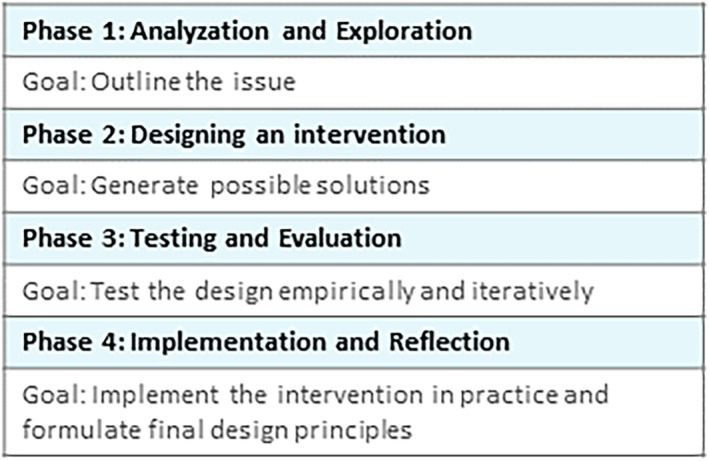
The four phases of design‐based research. [Color figure can be viewed at wileyonlinelibrary.com]

DBR comprises four distinct phases (Figure 2).^32^ During each phase, design principles are conceptualised and modified based on new data collected. Over the course of the DBR, an intervention that aligns with these design principles is developed and tested. DBR is characterised by triangulation, where data are collected through various research methods and sources to formulate design principles. The entire process unfolds within the context of practice and in collaboration with the stakeholders.

### 2.6 Data gathering and analysis

**FIGURE 3:**
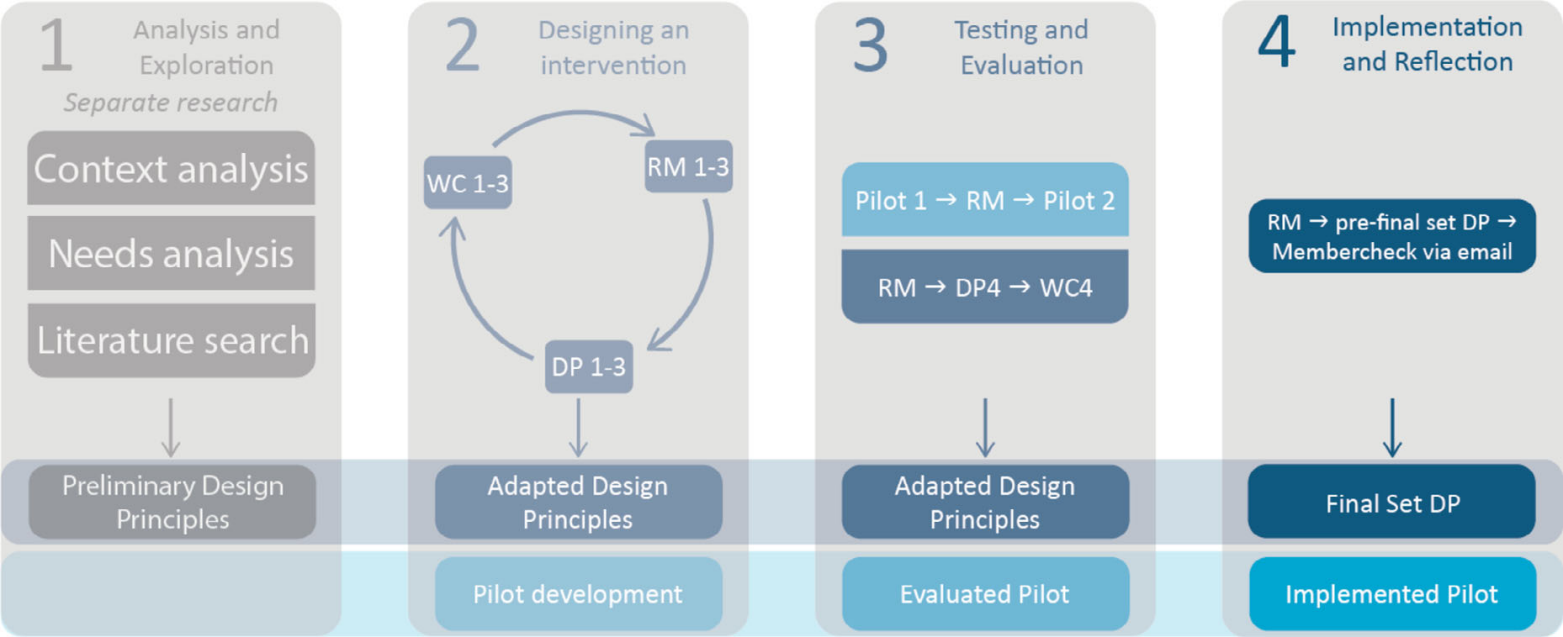
Design‐based research activities. WC = work conference; DP = design principles; RM = research meeting. [Color figure can be viewed at wileyonlinelibrary.com]

This DBR was conducted between September 2022 and October 2023. Due to the nature of DBR, the data gathering and analysis periods overlapped and will therefore be elaborated upon simultaneously. An overview of research activities can be found in Figure 3.

### 2.7 Phase 1: Analysis and exploration

Prior to the current study, we searched the existing literature and performed a needs and context analysis in a rapid ethnographic study with individual semi‐structured interviews and clinical observations.^8^ This mapped the current practice of PAL for clinical‐reasoning skill development among undergraduate students in the current workplace. This phase aided in the theoretic foundation and formulation of gaps and missed opportunities. Using the results of phase 1, a first set of preliminary design principles was formulated (Table 1; DP1). With these preliminary design principles, phases 2 through 4 commenced.

**TABLE 1:** The evolution of the design principles (DP).

DP 1	DP 2	DP 3	DP 4	Final set DP
The goal and potential of the PAL moment are clear to all stakeholders	The goal and potential of the PAL moment are clear to all stakeholders	Students and supervisors understand the purpose, importance, and potential of PAL	Students and supervisors understand the purpose, importance, and potential of PAL	0. Within the workplace, there is a culture focused on collaborative learning and development
			There is mutual respect and openness among students and supervisors	
Students are together (physically)	Students are together (physically)	Students are together (physically)	Students are together (physically)	1. Students are together (physically)
		Students feel they have ownership over PAL	Students feel they have ownership over PAL	2. Students are encouraged to take ownership of PAL
The roles of the various actors during the PAL moment are clear	The roles of the various actors during the PAL moment are clearly described	Students and supervisors are aware of and understand the various roles that can be taken in PAL	Students and supervisors are aware and understand the various roles that can be taken in PAL	3. Students have space to take on different roles in PAL
		PAL is integrated into the daily work process of the students	PAL is integrated into the daily work process of the students	4. PAL is facilitated as early as possible within workplace learning
			The context provides support for PAL in terms of space and time	
The supervisor is involved in the process at some point and only takes on the role of facilitator and/or content controller	The supervisor is involved in the process at some point and solely assumes the role of facilitator, initiator and/or content controller	Supervision can occur on content or process	Supervision can occur on content or process	5. Those involved actively question each other about learning outcomes on content and process as close as possible to the PAL moment
			Students can ask questions about their peer's clinical‐reasoning process in a constructive manner	
			Students and supervisor have tools to carry out the PAL activity effectively	6. Supervisors actively take a facilitating role in realising PAL

Abbreviations: DP, design principles; PAL, peer‐assisted learning.

### 2.8 Phase 2: Designing an intervention

Stakeholders were invited to participate in four WCs, each of which lasted 2 hours and was organised and chaired by LR, who provided preparation material when necessary. During these meetings, the stakeholders collaborated actively and creatively to generate ideas for potential interventions. As a form of triangulation, we asked for external input on the design principles in an open workshop organised at the annual conference of the NVMO. In this workshop (WC2), participants also worked on a rough sketch of an intervention.

Each WC started with a discussion on the latest version of the design principles (Table 1). The research group facilitated discussions during all WCs, observed and took notes on what was said. The outcomes were shared with the WC participants. Throughout these phases, the research group continuously reviewed and discussed the data alongside the design principles during various research meetings. These principles underwent iterative refinement based on newly acquired insights (Table 1).

### 2.9 Phase 3: Testing and evaluation

The prototype (Box 1) was tested during a 2‐month pilot at two locations: Radboudumc and CWZ. LR conducted all the evaluation sessions of the pilot. After the first month, the pilot was briefly discussed in a research meeting, and small alterations were made to the briefing students received beforehand. A summary of all pilot evaluations was made by LR, sent to the research group and discussed during WC4 and the research meeting afterward.

Box 1 **Paired consultation**

The core of the pilot involves students jointly conducting patient consultations at the outpatient clinic. Students receive a brief instruction at the beginning of their clerkship, including a pocket card containing general information on conducting a patient encounter with two individuals (Appendix 3). Two distinct roles are introduced: the executor and the observer. Each pair participates in at least two patient encounters, with each student alternating between the roles of ‘executor’ and ‘observer’. Students are responsible for preparing the patients either together or separately. Following the patient encounter, they collaborate on the patient's chart before meeting their designated supervisor to discuss the case. Subsequently, the supervisor and both students see the patient together to wrap up the encounter.

### 2.10 Phase 4: Implementation and reflection

All data were used by the research group to formulate a final set of design principles, which were checked by participants via an email round. Afterward, LR contacted both pilot locations and shared the final insights.

## 3 RESULTS

A total of 101 individuals participated in this study. Among them, 41 were students who took part in the pilot, 5 were supervisors involved in the pilot evaluation and 55 were stakeholders participating in one or more of the WCs. To maintain readability, we first describe the process by sharing data from the WCs (phases 2–3), followed by phase 4 including the final set of design principles.

### 3.1 Phase 2: Designing an intervention

#### 3.1.1 Work Conference 1

The purpose of this meeting was to diverge on various aspects of the topic. The initial part of the meeting focused on familiarising stakeholders with the theoretical background of clinical reasoning, PAL, DBR and the outcomes of phase 1. Using this knowledge, Set 1 (Table 1; DP1) of the design principles was thoroughly explored in smaller groups.

Several new topics were raised and discussed by stakeholders. For instance, it was noted that students might feel vulnerable when observed by their peers. Creating a safe learning environment in such situations would require both peers to be willing to be vulnerable. Other topics that arose were flexibility, autonomy, culture, reciprocity and unconscious incompetence. Additionally, the stakeholders expressed the belief that many students and supervisors are not fully aware of learning opportunities in the workplace, including PAL. They suggested that both students and supervisors would benefit from training to better recognise and utilise these learning moments. As the purpose of this meeting was to initiate the conversation, only minor modifications were made to the design principles (Table 1; DP2).

#### 3.1.2 Work Conference 2 (NVMO workshop)

During this workshop, 33 participants from diverse medical and educational backgrounds collaborated in smaller groups to devise solutions for promoting PAL in their respective workplaces, specifically aimed at the development of clinical‐reasoning skills in students. Following a brief introduction to the theoretical background, participants began by identifying key elements they deemed necessary for PAL to thrive.

Subsequently, they engaged in discussions and provided feedback on the proposed design principles (Table 1; DP2). The participants highlighted students' responsibility for PAL, which was consistent with the phase 1 interviews with supervisors, who advocated for student leadership in PAL.

Next, the participants of WC2 developed interventions to promote PAL for clinical‐reasoning skills development at their workplace. This resulted in 11 schematic interventions (Appendix 1). Worksheets used during the workshop were collected and utilised as data for analysis and the transformation of Set 2 into Set 3 of the design principles (Table 1).

#### 3.1.3 Work Conference 3

At this WC, the stakeholders built upon the discussion points and schematic interventions from WC1, WC2 and Set 3 of the design principles (Table 1; DP3). The stakeholders' conversation seemed to primarily focus on psychological safety in the workplace, including how to ensure it. Questions arose about students' need for connection in PAL activities and how competition might hinder PAL. While supervisors could foster psychological safety, a supportive, rather than controlling, attitude was preferred. Stakeholders also believed students should not have an active role in formal peer assessment, as it could impact psychological safety. Moreover, they acknowledged the connection between psychological safety and trusting peers' competence. Additional topics discussed in this meeting included creating ownership, establishing mutual respect between students and supervisors, and determining the level of explicitness of PAL activities. Four intervention blueprints were developed during this meeting as a result (Appendix 2).

#### 3.1.4 The pilot

Based on phase 1 of the DBR study, the notes of the previous three WCs and Set 3 of the design principles (Table 1; DP3), the research team developed a prototype PAL intervention (Box 1). The preferred intervention was one that could be seamlessly integrated into the workplace and daily activities of students, as the common belief was that when PAL becomes integrated into the work–learning space in clinical practice, it becomes part of normal practice for students. This was the reason our pilot PAL intervention was implemented in the first clerkship. To foster ownership, only guidelines were provided in the pilot. Students received brief instructions from LR in person and via e‐mail at the start of their clerkship, aimed at showcasing the potential of PAL and signposting the PAL opportunities in the workplace. At Radboudumc, students from the same group were randomly paired by the department's educational assistant. At CWZ, students had to self‐assign partners from a neighbouring student group. These differences were essential for implementing the pilot in their clerkship program. All supervisors of the pilot departments were informed via e‐mail about this pilot.

### 3.2 Phase 3: Testing and evaluation

#### 3.2.1 Pilot evaluation

Overall, the pilot was considered a success by all involved and a valuable addition to other (individual) clinical‐reasoning learning activities in the workplace (Q1). For example, students observed and talked about differences in the questions their peers asked during consultations, which influenced differential diagnoses. Additionally, we observed that the number of patient encounters per student increased by a factor of 1.5 due to the paired consultations.

‘Regarding clinical reasoning: there was a lot of discussion about the patient. For example, they talked about separating the main issues from secondary ones (what is relevant and what is not). They also cross‐checked their findings from the physical examination to see if the other person had noticed the same things’.—student evaluation note

The setup for the paired consultation in this pilot allowed students to develop ownership of their PAL. Once an initial PAL session was scheduled, students readily embraced their responsibility, showed enthusiasm and took the initiative to organise more PAL sessions themselves, creating a collaborative environment during their clerkship (Q2). Students mentioned their intention to use paired consultation in future clerkships as well, depending on the perceived space to organise their own learning activities. Moreover, students expressed a preference for follow‐up sessions with their peers to monitor their growth, which can be helpful in comparing individual progression.

‘Because the paired consultation sessions were scheduled in the timetable, the students were able to find each other quickly and easily when something was cancelled or when they had free time. They would ask around to see if they could join someone at the last minute, and they did so’.—student evaluation note

During the pilot, the students were given a pocket card that introduced them to two roles for their PAL activity: executor and observer (Box 1). While many carried the pocket card throughout the day, some students did not read the content. Those who had not read it later found the information valuable but suggested a different format. Some naturally assumed the suggested roles, while others misunderstood them, thinking an observer should remain passive during the consultation. Conversely, other students chose to actively participate in patient encounters, with one of them taking the lead, resulting in more collaborative learning (Q3).

‘The executor was mostly speaking, but they regularly involved each other in the conversation. More often, they also used the ‘we’ form to refer to their actions: ‘we did this/looked at …’. […] In the debrief, the [supervisor and two students] also conducted the conversation together. […] The students regularly complemented each other. [Supervisor and two students] also performed the physical examination together on the patient’.—supervisor evaluation note

Throughout this research, various supervisors shared personal breakthroughs. For example, as they gained knowledge about its purpose and potential, they expressed growing enthusiasm for using PAL to develop student clinical‐reasoning skills in practice. They shared that they themselves use PAL daily but confirmed that students are rarely present during those times. They acknowledge their task as role models towards students in making PAL more explicit in their own practice. Before this study began, it was assumed a supervisor should always be present to initiate or guide the clinical‐reasoning learning process and that it was a time‐consuming endeavour. Fortunately, the pilot proved some students were perfectly able to start this by themselves. The overall time required was not substantially shortened or lengthened for supervisors; it was filled differently with more in‐depth discussions (Q4 and Q5).

‘It does not take more time; in fact, it may take less because you can explain the background of an illness to two students at once. As a result, both students will understand the illness better when they encounter a patient with the same problem again’.—supervisor evaluation note

‘Having two students present leads to more discussion during the debriefing (in a positive way)’.—supervisor evaluation note

#### 3.2.2 Work Conference 4

Before WC4 commenced, the research group used the results from previous WCs and the pilot evaluations to formulate Set 4 of the design principles (Table 1; DP4). During the fourth and final WC, all stakeholders extensively reflected on both the pilot and the design principles. LR shared the summary of the pilot evaluation sessions with all stakeholders during an interactive group conversation, enabling stakeholders to provide immediate reactions, questions and remarks.

Following this conversation, the remaining time of the WC was dedicated to a detailed evaluation of each design principle, focusing on both its meaning and formulation. Throughout this WC, participants realised that certain topics (e.g. psychological safety, making PAL explicit to students) represented a particular culture and that this would form the basis for learning in general (design principle 0).

### 3.3 Phase 4: Implementation and reflection

Both locations are continuing paired consultation in their clerkships, and LR helped them to adjust it to better fit their context using research results. A wallposter about paired consultation was created that can be put in a dedicated student room, replacing the pocket card. One stakeholder who participated in the WCs consulted LR to explore the possibility of starting a pilot in their own context. Below, in Table 2, the final design principles and their descriptions are presented.

**TABLE 2:** Final set of design principles and their description.

Design principle	Description
0. Within the workplace, there is a culture focused on collaborative learning and development.	For students to effectively learn, it is crucial to cultivate a culture that supports their educational journey. This culture should also involve leading by example, with students observing experienced individuals engaging in learning activities within the workplace to understand their relevance to future professional development. The visible involvement of residents and medical specialists in PAL activities is essential for students to perceive PAL as integral to their own learning. Creating a psychologically safe environment in the workplace is paramount for implementing PAL as a learning strategy. This underscores the importance of fostering a non‐competitive atmosphere among students and the various professionals working within the clinical setting.
1. Students are together (physically).	For PAL to take place, students need to be together. This is especially crucial in the workplace where students are developing their clinical‐reasoning skills. While physical proximity is essential for PAL to flourish, nonphysical forms of contact such as phone calls or video conferences could also be helpful. However, if students are not brought together or do not have a designated shared space, PAL activities are unlikely to occur; therefore, physical presence continues to be preferred.
2. Students are encouraged to take ownership of PAL.	Students often struggle to take ownership of PAL at the beginning of their clerkships because they are unfamiliar with the opportunities available at their clerkship location. They may not feel empowered to organise PAL activities and need help to do so. Active encouragement from supervisors regarding the various opportunities for PAL in the workplace, particularly for clinical‐reasoning development, greatly motivates students to engage in PAL activities. Scheduling an evaluation session, as done in the pilot, was beneficial in encouraging students to take ownership of PAL.
3. Students have space to take on different roles in PAL.	Students should be familiar with the various roles in PAL and feel free to take on the roles as they see fit to facilitate learning. Not being able to do so may lead to learner regression.
4. PAL is facilitated as early as possible within workplace learning.	It is essential to design a workplace that facilitates PAL, which could involve creating a dedicated student room or other special contexts for specific learning interventions. During the early stages of clinical clerkships, students primarily utilise PAL for role modelling and to familiarise themselves with the logistics of clinical clerkships and intern responsibilities. As they progress, they become more adept at recognising and discussing style differences with their peers, leading to meaningful reflections and enhancing their (meta)cognitive clinical‐reasoning skills. Follow‐up sessions can be helpful to monitor individual progression.
5. Those involved actively question each other about learning outcomes on content and process as close as possible to the PAL moment.	Feedback can focus on both content and process. Content feedback aims to articulate the knowledge gained from the PAL session, while process evaluation involves assessing how students conducted the PAL activity and encourages the development of metacognitive skills, particularly ‘learning how to learn’. Questions such as ‘What have you learned from this?’, ‘What were some effective questions posed by your peers?’, ‘How did you find collaborating on this task?’ or ‘What insights did you gain from each other?’ can facilitate this reflective process. Ideally, feedback should be provided as soon as possible after the PAL session while the experience is still fresh in their memories. This can be initiated either by the students themselves or by a supervisor. For the moments when students need a guiding hand in practice, supervisors need tools to instigate this process among students. As supervisors are sometimes witnesses of PAL moments, and PAL demonstrates its fullest potential in a collaborative learning environment, they can also be involved in guiding questions. While formalising feedback in a portfolio is an option, students generally prefer an informal approach to avoid altering group dynamics.
6. Supervisors actively take a facilitating role in realising PAL.	Supervisors play a delicate role in PAL. They often perceive themselves as the primary source of knowledge (‘truthtellers’) in learning activities. Despite their good intentions, supervisors may tend to dominate conversations instead of facilitating deep discussions among peers and providing occasional guidance from the background. While they are essential for ensuring the accuracy of the knowledge acquired by students, they must also adopt a passively active approach during PAL activities to truly empower PAL in the clinical‐reasoning learning process.

Abbreviation: PAL, peer‐assisted learning.

## 4 DISCUSSION

This study explored what is needed to create a clinical learning environment that fosters PAL to enhance the clinical‐reasoning learning process for undergraduate students. The key points revolve around the responsibility of students, the role of supervisors and the creation of a collaborative learning environment. Alongside the development and implementation of an intervention to stimulate this environment, design principles were formulated that can be used to design interventions in other contexts (Table 2).

Despite recent literature stating that PAL should be more about learning *with* peers rather than learning *from* peers,^3^ it remains challenging to put this mindset into practice. Within this research, we observed that students, when in the observer role, sometimes felt less responsible for patient encounters when another student was the executor, resulting in more passive learning behaviour. This shows that, even in a situation among two same‐level peers, a derived thought of inequality in learning roles is present. We hypothesise that this is related to the cognitive apprenticeship model that medical students are familiar with when entering practice,^12^, ^25^ in which students are accustomed to a hierarchical role distribution in clinical learning settings rather than an egalitarian learning relationship. While our research shows it is hard to breach this, we fortunately also saw students actively collaborate on patient encounters despite being appointed as executor and observer. We therefore believe that it is imperative to start to stimulate favourable learning attitudes early in a student's clinical learning experience and to remove any unwanted seeds before they grow into undesirable learning behaviour.

The role of the supervisor in students' PAL for the development of clinical‐reasoning skills remains a delicate one, but supervisor input and guidance are needed for PAL to be successful in the workplace. As medical students enter clinical practice, they are introduced to specific communities of practice where they continue their education.^12^, ^34^, ^35^ Being newcomers, they must adapt to the established rules and norms; however, their limited familiarity with the community also means they lack insight into which learning activities are feasible and beneficial. They need supervisors, the people who are positioned more towards the core of the community, to show and guide them towards their role as clinical learners. Explicitly highlighting the need for change towards collaborative learning practices for students to supervisors can be a significant first step.

When students enter clinical practice, they step into a community of practice with the medical specialist at the centre. Coming from the medical faculty, where the centre of the community was formed by the learners,^36^ they need to acclimate to this new learning environment. Earlier research showed that in this transition, the community of learners might dissolve into the community of practice, leaving the role of peers in an uncertain state.^8^ As PAL is particularly useful to clinical learners, we were enlightened to see that this research seemed to spark a revival of the community of learners within a community of practice. This makes us feel optimistic for a future in which collaborative learning in the workplace is possible using PAL for the development of clinical‐reasoning skills.

For the health profession's education community to move towards this collaborative future, further research is needed. Potential research ideas include focusing on the community of learners within the community of practice and exploring how they can strengthen each other further in the development of clinical‐reasoning skills. Another angle would be to investigate how PAL can contribute to inter‐ and intra‐professional learning behaviour, with which PAL is often associated.^37^, ^38^ Additionally, research could explore the impact of PAL on the outcomes of the clinical‐reasoning process and examine which aspects of PAL become a permanent part of physicians' professional lives. Finally, and arguably, the most challenging is to investigate how we can guide the workplace, and everyone involved, through the cultural changes necessary for future learning, considering these individuals were brought up in a different system.

### 4.1 Strengths and limitations

Conducting DBR comes with some limitations. We chose a relatively short period for testing the pilot. Although a longer period could provide more development time and yield additional (quantitative) evaluation data, we found that the data collected were already rich and sufficient for our DBR process. Additionally, the pilot evaluations were conducted at the end of the clerkship, which means the students' thoughts could be influenced by hindsight bias. Ideally, students should be interviewed immediately after a paired consultation. Lastly, a common limitation of DBR is its transferability to other contexts. A strength of our study was our approach to enhance transferability, in which we formulated overarching design principles and discussed them with various stakeholders from different contexts, frequently comparing them with existing theories. Other strengths of our methodology include the involvement of a broad group of stakeholders who actively participated in the discussion and evolution of the design principles, not just of the pilot development.

## 5 CONCLUSION

This research investigates what is necessary to create a clinical learning environment that invites PAL for the development of clinical‐reasoning skills. A set of design principles was formulated to develop interventions for various contexts. To ensure these interventions are successful, three topics deserve attention: (1) Students must feel equally responsible for learning *with* peers rather than *from* peers, (2) supervisors must contribute to the evolution towards a collaborative learning environment for the enhancement of the clinical‐reasoning learning process and (3) the formation of a community of learners within a community of practice might enhance the collaborative learning practice. Further research should focus on guiding the workplace in this transition towards collaborative learning.

## AUTHOR CONTRIBUTIONS

**Larissa I.A. Ruczynski:** Conceptualization; investigation; writing—original draft; methodology; validation; visualization; writing—review and editing; software; formal analysis; project administration; data curation; resources. **Bas J.J.W. Schouwenberg:** Supervision; writing—review and editing; formal analysis; methodology; investigation; conceptualization. **Cornelia R.M.G. Fluit:** Supervision; writing—review and editing; formal analysis; methodology; investigation; conceptualization. **Marjolein H.J. Pol:** Supervision; writing—review and editing; formal analysis; methodology; investigation; conceptualization.

## CONFLICTS OF INTEREST

The authors declare no conflicts of interest.

## Supporting information


**Appendix S1.** 11 schematic interventions from WC2 (NVMO).

**Appendix S2.** 4 intervention blueprints from WC3.

**Appendix S3.** pocket card used during the pilot intervention.

## Data Availability

The data that support the findings of this study are available from the corresponding author upon reasonable request.
