# Supplementary material for: The power of peers: Design‐based research on stimulating peer‐assisted learning for enhancing the clinical‐reasoning learning process in the workplace
Source: Med Educ. 2025 Feb 14;59(7):739–49. doi: 10.1111/medu.15613 (PMC12198721; doi:10.1111/medu.15613)
Supplement: Supplementary file 1 — Appendix S1. 11 schematic interventions from WC2 (NVMO). [file MEDU-59-739-s003.docx]

**Appendix 1: 11 schematic interventions from WC2 (NVMO)**

NOTE: This is translated from Dutch.

During this workshop, we had four interactive assignments. For the completion of these assignments, we have asked the participants to fill out a worksheet. The four assignments were:

1. According to you, what is essential for learning with/from other people? Out of the words you have written down, what is the keyword?
2. We did a short presentation on background theory, phase 1 of this research and DP1 (see Table 1). We asked them: would you like to change anything about the design principles? If so, please share what you would like to change here. Do you want to add something or leave anything out?
3. Ideas for possible interventions that meet the design principles.
4. Development of a schematic intervention. To do this, we asked them to provide the following information:
   1. Title of the intervention
   2. Target group
   3. Roles of participants
   4. Content of the intervention (what steps are involved)
   5. Why do you think this will work?

Below we list the 11 schematic interventions that came out of this session.

1. Patient case ‘patient unsolved’
2. Target group: essentially anyone and everyone
3. Roles of participants: assessing learning objectives and guiding from the background (or not).
4. Content of the intervention:
   - 1. Outpatient clinic and wards.
     2. Logistics, location, supervisors, numbers, group size
     3. Follow-up (presentations?)/learning goals/exploring
     4. How to go from diagnosis to therapeutic path (‘methodology’)
     5. ‘Playing doctor’
     6. How to assess?
5. Why do you think this will work?
   - 1. Not done after first [undecipherable word] when ‘supervisor’ spoon-feeds everything.
     2. Learning with and from each other!!!
     3. ‘Interdisciplinary’
6. Alternatives: dual-learning medical intern
7. ‘Co-kwartier’ (meaning 15 min. dedicated to the medical interns every day after morning- and afternoon handover)
8. Target group: medical interns at a ward during 1^st^ and 2^nd^ clerkship (year 1) and their elective (year 1-3) and senior clerkship (year 3).
9. Roles of participants:
   - 1. Medical interns collaborate to select a problem based on the morning handover or their recent experiences in the workplace.
     2. Supervisor (resident/medical specialist) facilitates the discussion, asks questions, and encourages reasoning.
10. Content of the intervention:
    - 1. Scheduling the ‘co-kwartier’
      2. Reserving a space
      3. Creating a group of enthusiastic supervisors
11. Why do you think this will work?
    - 1. We’re already doing it! Students highly appreciate the approach
      2. Students learn from one another
      3. Minimal time required from the supervisor
      4. Students build rapport, making it easier to connect with each other at the workplace
12. DUO-learning
13. Target group: medical interns in general practice
14. Roles of participants:
    - 1. Medical intern visits a peer’s practice and vice versa.
      2. General practitioner/supervisor guides and supervises the interns.
15. Content of the intervention:
    - 1. The intern spends two days shadowing a peer in another practice/workplace.
      2. Additional element: feedback is provided by a different supervisor.
16. Why do you think this will work?
    - 1. Interns gain exposure to diversity in general practice settings and supervisors.
      2. Additional benefit: different feedback from another supervisor.
17. Interprofessional peer learning
18. Target group: interprofessional morning briefing for students without a supervisor.
19. Roles of participants: supervisor/workplace mentor creates and protects time for this activity.
20. Content of the intervention:
    - 1. After the morning handover, students set individual and paired/group learning goals.
      2. Share significant learning moments with the group.
      3. Agree on which students can provide guidance on specific learning goals.
21. Why do you think this will work?
    - 1. Provides students with insights into the focus areas of various healthcare training programs (interprofessional).
      2. Establishes a safe learning environment.
      3. Encourages students to function as a team.
22. Duo-consultation
    1. Target group: Master’s students (during clinical clerkships) from all years of the master’s program.
    2. Roles of participants:
       1. 1^st^ and 2^nd^ year Master’s student pair up to form a duo.
       2. Medical specialist/supervisor conducts a meeting afterwards and provides feedback.
    3. Content of the intervention:
       1. Duo consultation (pre-established learning goals are formulated).
       2. Examining a patient together as a pair.
       3. Opportunity for immediate peer consultation.
       4. Acting as a check for each other.
       5. Brainstorming and reflecting together.
       6. Structuring the handover to the supervisor.
       7. Providing feedback to the patient.
    4. Why do you think this will work?
       1. While it may not suit every situation, it offers significant support, especially for beginner medical interns. The responsibility for the patient is shared.
23. Morning start and end-of-day reflection
    1. Target group: medical interns and nursing students in the workplace.
    2. Roles of participants: [left blank]
    3. Content of the intervention:
       1. Morning start (5 min. before rounds): students discuss what they want to learn that day. This is an unstructured session, but time and space must be allocated for it. Setting and discussing learning goals among peers.
       2. End-of-day reflection (15 min. after handover): guided by a process facilitator (non-content-specific) or sometimes a content-focused supervisor. The discussion revolves around what students learned during the day. Reflecting on the learning goals set earlier in the day, reviewing what was learned, and verifying this with a content supervisor.
    4. Why do you think this will work?
       1. Time is specifically allocated for thinking about personal learning goals and reflecting on them.
24. Physician assistants (PA)
    1. Target group: physician assistants learning in their own workplace with fellow PAs
    2. Roles of participants: Facilitate informal exchanges of questions and experiences with peers who are not in a supervisory or evaluative role.
    3. Content of the intervention: currently brainstorming
       1. Involve supervisors but more as peers – for example, working through cases together and presenting them.
       2. Create opportunities for Pas to connect and exchange experiences with peers from other workplaces.
    4. Why do you think this will work? [left blank]
25. Croquette lunch
26. Target group: medical interns (with an appetite) and residents (not) in training
27. Roles of participants: eat and engage in discussions. Each group (resident not in training first, resident in training second and interns third) contributes to the conversation.
28. Content of the intervention:
    - 1. Discuss a medical ethical dilemma over a croquette lunch.
      2. Both residents not in training and interns contribute personal dilemmas, particularly moments where they doubted their actions or made a mistake.
      3. Ideally, incorporate a humorous element (e.g., sharing a funny case).
29. Why do you think this will work?
    - 1. Encouraging residents (not) in training to take the lead fosters vulnerability among peers.
      2. Informal setting.
      3. Safe environment, as medical specialists are not present.
30. Interprofessional - patient
31. Target group: Students from various fields/disciplines working together with a patient.
32. Roles of participants: each participant contributes from their specific professional perspective.
    1. Develop a care plan.
    2. Address patient-related questions.
    3. Focus on ‘learning together’, understanding each participant’s focus area while benefitting from shared knowledge and insights.
33. Content of the intervention:
    1. Analyze the patient’s questions.
    2. Examine the situation from different perspectives.
    3. Work together to reach a conclusion or recommendation.
    4. Emphasize collaboration and mutual learning.
34. Why do you think this will work?
    1. Provides insight into each other’s expertise and thought processes, enabling better advice.
35. Teaching for medical interns
36. Target group: medical interns in a large regional hospital (around 80 interns).
37. Roles of participants: two medical interns and a medical specialist (supervisor)
38. Content of the intervention:
    1. two medical interns prepare and deliver a lecture, supervised by a supervisor.
39. Why do you think this will work?
    1. Involves supervisors
    2. Smaller groups enhance focus without necessarily increasing workload (provided clear frameworks are established).
40. Communication skills
41. Target group: [left blank]
42. Roles of participants: [left blank]
43. Content of the intervention:
    1. Three safe preparatory moments, followed by assessment through self and peer feedback.
    2. (1) First, how did the student think it went? Second, feedback from simulated patients. Third, feedback from peers. Fourth, feedback from instructor. It is mandatory for students to actively question each other.
    3. (2) ‘Playground environment’. Multiple sessions leading up to a final assessment or proposal. In the ‘playground’, instructors adopt a more reserved role. Provides space for students to make and learn from mistakes.
    4. (3) Case-based discussion: groups of four students discuss a provided case with an instructor present.
44. Why do you think this will work? [left blank]
