# Supplementary material for: The power of peers: Design‐based research on stimulating peer‐assisted learning for enhancing the clinical‐reasoning learning process in the workplace
Source: Med Educ. 2025 Feb 14;59(7):739–49. doi: 10.1111/medu.15613 (PMC12198721; doi:10.1111/medu.15613)
Supplement: Supplementary file 2 — Appendix S2. 4 intervention blueprints from WC3. [file MEDU-59-739-s002.docx]

**Appendix 2 – 4 intervention blueprints from WC3**

NOTE: This is translated from Dutch.

**Design 1**

*Title:* [left blank]

*Target group:* medical interns from Master’s year 1 and 2

*Placement of the intervention in the Master’s curriculum: practical setting*

*Time required for the intervention:* Varies by discipline, between 1-3 hours.

*Preparation needed:*

- Set up an educational outpatient clinic and provide an incentive for patients (e.g., shorter waiting times).
- No specific preparation required.
- Ensure departmental support for implementation.
- In the bachelor’s phase: practice working in pairs so interns are already familiar with and aware of PAL concepts.

*Description of the intervention:*

- Pairs of students prepare for the patient encounter.
- Take turns conducting consultations.
- Reflect on clinical reasoning and formulate follow-up plans
- Conclude with supervision.

*Additional notes:*

- Important to provide variation in approach.
- Allow participants some flexibility in how they conduct the intervention.
- In year 1, emphasize implementation and familiarity. In year 2, encourage autonomy and ownership.
- In cases where only one intern is available in a hospital, use neer-peer assisted learning.

*Role of the participants* [left blank]

*Why do you think this works?*

- Prepares students for discussion with their supervisors (‘spin-off’)
- Increases self-efficacy
- Aligns with all design principles
- Reflects authentic situations with professional responsibilities.
- Involves real patients, adding pressure to perform well.
- Encourages knowledge-sharing.
- Motivates participants to succeed.
- Facilitates critical reflection.
- Offers additional insights through peer feedback.
- Enhances communication skills.
- Expands practical skill repertoire.

*Potential pittfalls*

- *Non-compliance by participants*
- *Risk of it becoming a routine task without engagement (‘checkbox mentality’).*
- *Logistical or departmental challenges in providing adequate facilities.*

**Design 2**

*Title:* Prepare your own patient together (PYOPT)

*Target group:* suitable for all phases of education within the hospital. Applicable wherever there are at least two interns.

*Placement of the intervention in the Master’s curriculum:* during clinical clerkships, applicable to any clerkship.

*Time required for the intervention:* one hour daily, prior to the afternoon or evening handover.

*Preparation needed:*

- A reserved room, ideally a dedicated student working room 😉
- One hour of time
- Access to a sufficient number of computers.
- Knowledge of which patients they will see the next day.
- Ten minutes of the supervisor’s time, scheduled in advance.

*Description of the intervention:*

- One hour before the handover, interns sit together in a room to prepare for the patients they will see the next day (either in the ward or outpatient clinic). A suggested schedule is provided for their use but is optional.
- First 10 minutes: reflection on the previous day – what worked, what they learned, and key takeaways.
- Next 40 minutes: preparation for the following day.
- Last 10 minutes: The supervisor (resident/medical specialist) joins for a discussion.
- Note: interns decide among themselves how to allocate the patients.

*Role of the participants* Refer to the schedule described above.

*Why do you think this works?*

- It ensures everyone is in the same place at the same time.
- It builds on an existing, informal practice by formalizing and facilitating it.
- Preparation for the next day is already completed.

**Design 3**

*Title:* PALI (instead of ‘poli’, the Dutch abbreviation used for outpatient clinic) / running a clinic together / alternating consultations

*Target group:* interns at any stage of their training. Possibly also pairs of two consecutive groups (e.g., 4-week/8-week rotations).

*Placement of the intervention in the Master’s curriculum:*

- Suitable for all clinical clerkships.
- Encourages placing more interns on the same department.
- Second-best option: interns visit each other’s placements if only one intern is assigned to a department.

*Time required for the intervention:* Fully integrated into the clinical workflow. Does not extend the length of the clerkship or supervision time.

*Preparation needed:*

- Normal clinic preparation.
- Interns should alternate roles multiple times, including observing and taking the lead.

*Description of the intervention:*

- Intern-run outpatient clinic: conducted alternately by two interns.
- Patient cases are reviewed with one supervisor.
  - Learning outcomes from working together are discussed separately (without the supervisor initially), followed by the supervisor later asking, ‘How did it go’? This discussion includes what went well and what could be improved.
- Clinical reasoning is actively reviewed, using tools like the clinical reasoning cycle if necessary.

*Role of the participants:*

- Interns need to clearly formulate their own learning objectives.
- Variations in learning objectives among interns are acceptable and encouraged.

*Why do you think this works?*

- Exposure to more patients
- Insight into how peers handle clinical cases.
- Encourages a collegia land enjoyable environment.
- Stimulates asking questions.
- Keeps the patient at the center of the process.
- This initiative promotes ownership and engagement among interns.

**Design 4**

*Title:* Reflection on PAL (on a formal education day – FED)

*Target group:* Interns attending a FED at the university focused on clinical reasoning.

*Placement of the intervention in the Master’s curriculum:*

- Every intern has seen patients, so each van present a case.
- Consideration could be given to systematically discussing clinical reasoning within the PAL framework.

*Time required for the intervention:*

- This can take 1-2 hours.
- Can be conducted either in person or digitally.
- Involves part of the student group in rotating subgroups (e.g., digital breakout rooms).

*Preparation needed:*

- Minimal, as all participants have seen patients.
- Ideally conducted every two weeks for two hours, ensuring everyone gets a turn over time.
- Additional benefits: context-sharing, peer support, and bridging academic and peripheral case discussions.

*Description of the intervention:* refer to details above.

*Role of the participants:* group engagement through smaller subgroup discussions.

*Why do you think this works?* Refer to details above.
