# Supplementary material for: The power of peers: Design‐based research on stimulating peer‐assisted learning for enhancing the clinical‐reasoning learning process in the workplace
Source: Med Educ. 2025 Feb 14;59(7):739–49. doi: 10.1111/medu.15613 (PMC12198721; doi:10.1111/medu.15613)
Supplement: Supplementary file 3 — Appendix S3. pocket card used during the pilot intervention. [file MEDU-59-739-s001.docx]

**Appendix 3 – pocket card used during the pilot intervention**

Students print and fold this page so that it eventually becomes a small booklet with all the text in the right way.

**
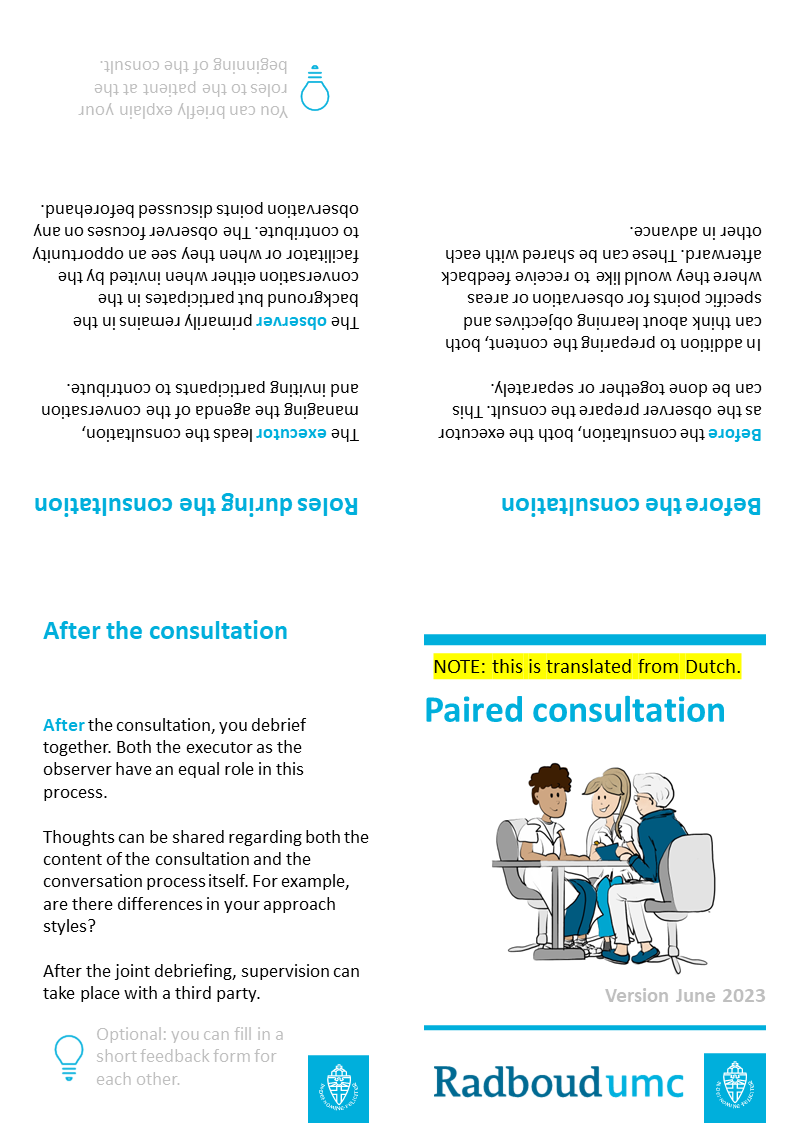
**
